# Redundant packets removal queue-aware congestion avoidance scheme for IoT based healthcare applications

**DOI:** 10.1186/s12962-026-00737-w

**Published:** 2026-03-23

**Authors:** Muhammad Zafarullah, Ata Ullah, Sajjad A. Ghauri, Nauman Anwar Baig

**Affiliations:** 1 Department of Computer Science, National University of Modern Languages (NUML), Islamabad, 44000 Pakistan; 2 School of Engineering & Applied Sciences, Isra University, Islamabad, Pakistan; 3 School of Computing, Engineering and Technology, Robert Gordon University, Aberdeen, UK; 4 SEECS, National University of Science & Technology, Islamabad, Pakistan

**Keywords:** Patient-centric IoHT, SVM, ANN, Smart devices, Congestion control

## Abstract

The Internet of Healthcare Things (IoHT) is an emerging technology that has attracted researchers’ interest due to its broad applicability in recent years. In IoHT platforms, sensors continually collect patient data, which most of the time contains normal readings, and send it to the cloud via intermediate devices. In an emergency, a large amount of data may rapidly fill network queues, causing congestion and data loss. It has significant consequences, particularly in time-sensitive healthcare applications. To solve this issue, the suggested approach minimizes data transfer by transmitting a Boolean value if no emergency is identified or the patient’s health parameters are normal. The aforementioned Boolean value is far less in size and takes much less time to process data, significantly decreasing the cache’s burden and freeing it up for unforeseen emergency data. This approach aims to improve the efficiency of packet data handling. This ensures that high-priority, critical data packets are processed immediately. Furthermore, after transferring 20 packets via Boolean values, the system then sends the entire data packet to keep track. This balanced strategy reduces unnecessary data traffic, alleviates network congestion, and ensures that vital information is processed on time. By reducing cache use and prioritizing emergency packets, the suggested solution improves the overall performance of the IoHT system and enhances patient safety by preventing the loss of critical data. A large number of simulations are performed on Ubuntu using NS-2.35. The results show that the proposed scheme outperforms in terms of communication cost, throughput, packet loss, and delay during an emergency.

## Introduction

The Internet of Healthcare Things (IoHT) comprises wearables and non-wearable devices, sensor/actuator circuits, wireless communication technologies, remote health monitoring, and IoT tools and applications [1, 2]. IoHT creates a sophisticated healthcare system that connects many components to offer users a broad range of smart solutions [3]. IoHT enables interaction between humans and machines and real-time monitoring of health systems, hence increasing patient engagement in decision-making. Major benefits of IoHT systems include lower healthcare costs, real-time responses to emergencies, and remote monitoring during pandemics. The growing significance of IoHT stems from current innovations in remote health, smart detection, and medical monitoring. IoHT devices can communicate with healthcare providers and medical professionals using online servers [4].

Patients’ vital health data is often processed briefly before being sent to a central body [5]. Medical specialists have access to the data and can study it at any time and make the appropriate decisions at the correct moment [6]. Data aggregation involves collecting, combining, and summarizing data at an aggregator node before forwarding it to other nodes. IoT-enabled WSNs can operate in both homogeneous and heterogeneous environments. A homogeneous network is easier to manage because all nodes generate similar data. The heterogeneous ecosystem is highly difficult since nodes vary and generate data in different formats [7]. Data aggregation methodologies vary depending on network size. The most prevalent data aggregation strategies are centralized (CDA) and in-network (IDA). The CDA is frequently used in small networks having just one collector that gathers data from each node. The strategy used by IDA improves the previous technique by deploying several AN nodes throughout the network to gather and transmit data to a Base Station [8].

De-duplication is a technique for efficiently managing large amounts of data by removing duplicates [9]. The de-duplication method involves two primary strategies. Inline de-duplication is a real-time process that checks data for duplication before storing it. Background de-duplication is a post-processing step that occurs after data has been written to storage. Duplicates are identified and removed from current data by periodic scanning [10]. The healthcare sector’s extensive gathering of health records, diagnostics reports, and histories of patients may lead to redundant data, resulting in increased storage space and energy usage. Repeated data degrades sensor device resources and directly correlates with energy consumption and network lifetime [11].

Developing innovative techniques in healthcare is crucial for continuously improving and innovating medical monitoring systems. Healthcare schemes encounter difficulties such as insufficient data aggregation, duplicated data handling, excessive energy use, and cost of storage in diversified locations [12]. Sensor equipment in healthcare acquire and transmit detailed data to BS on a regular basis. Excessive data replication consumes sensor nodes’ limited resources [13]. Use these resources with caution, especially in healthcare, where accurate information is critical for effective treatment. The proposed approach is critical for successfully addressing these issues.

IoHT generates vast amounts of health data, which are stored in local or globally cloud repositories that hospital employees, physicians, and caretakers can access. It facilitates prediction, correlation, and classification by analyzing test results, medication, symptoms, and healthcare indicators tracked throughout the day [14, 15]. Through an IoHT network, smart devices exchange, transmit, and display medical data. The large number of sensing devices may cause network congestion, which leads to low throughput, excessive energy consumption, and packet loss. A large volume of messages sent during an emergency, which could lead to congestion. If the sender is not informed of the queue status of neighboring nodes, the issue becomes worse.

The emergence in IoHT has led to the integration of multiple IoT devices with the patient’s body, increasing the volume of requests that can cause congestion. Communication could be delayed as a result, and the most important request might not be handled promptly. Although the issue is resolved by the Q-based prioritization techniques, the sender node still faces difficulties in detecting congestion early because neighboring nodes do not communicate the queue’s state. The difficult challenges in IoHT are recognizing congestion early, being aware of nearby nodes, and responding quickly to emergency packets.

The main contribution of this paper is as follows;

1. We reviewed the literature on queue-aware congestion-avoiding approaches. We identified a significant research gap in handling sensor data during emergencies, where cache congestion can cause the loss of critical packets, affecting healthcare outcomes.
2. The study then presents a unique scheme for handling IoHT data transmission, in which non-emergency data is delivered as Boolean values to alleviate congestion caused by cache overflow, and full data packets are transmitted after 20 Boolean packets for record-keeping or when there is a change in the sensor data reading. This contribution significantly improves the effectiveness of emergency data handling by reserving cache space for vital information, thereby enhancing the overall dependability and response of IoHT systems during emergencies.
3. Extensive simulations are performed to validate the proposed scheme and compare its performance to prevailing base schemes.

The rest of the work has been organized as follows; Sect. 2 explores the literature on data aggregation in IoHT networks, and processing critical packets w.r.t health monitoring system. Section 3 discusses the system model with problem statement. Section 4 focuses in the simulation and its results. Finally, Sect. 5 outlines the conclusion and next steps.

## R{2,5} Motivation of the research

**R{1,2} The motivation of this study arises from the rapid growth of IoHT implementations, which generate large volumes of patient data that require effective, timely delivery. Under such systems, there is unnecessary pressure on network bandwidth, buffer capacity, and energy resources due to frequent exchanges of redundant and stable physiological measurements, leading to congestion, packet loss, and delayed delivery of vital information in emergencies. These problems are especially acute in resource-limited medical facilities and the monitoring of vast populations. Therefore, this study is motivated by the need to develop a cost-conscious and resource-efficient congestion-avoidance strategy that will curb unnecessary data transmission whilst ensuring the reliable delivery of emergency and clinically relevant information. This mechanism would boost the overall efficiency and robustness of IoHT’s communication systems.**

## Literature review

This section discusses current research conducted in the field of IoHT and its applications. The research society has recently expressed a considerable interest in IoT-based healthcare solutions. Thus, congestion avoidance or management is a demanding study topic in IoHT field.

The author in [16] proposed utilizing a fuzzy matrix to transport data from IoT devices to a backend system. The gathered data is then forwarded into the edge server. The cloud server monitors the edge server and using blockchain technology to avoid malicious attacks. Sensor nodes transmit encrypted information to aggregating nodes. To reduce energy, bandwidth, and storage costs, Ans delivers compact information to the fog servers [17]. Ananth in [18] used glow swarm optimization to cluster data. The proposed technique is ideal for medical applications due to its lower latency. However, multi-layer clustering may add complexity.

In [19], A fog-based computational framework and its potential applications in the Internet of Things are proposed. It has a large capacity to evaluate data nearer to the source, reducing latency and improving reaction times. Its capabilities includes distributed processing, improved safety, and scalability, making it perfect for real-time applications in healthcare. Despite these benefits, challenges such as managing resource constraints, ensuring security of data, and optimizing power consumption remain. Addressing these issues is crucial to properly implementing fog computing in medical centers.

The fuzzy-based data aggregation approach is proposed to improve energy efficiency in healthcare. To address the various circumstances, each node gets assigned an appropriate parent node. The data is then gathered and checked for duplicate entries. If these values exist, they get substituted with the Boolean value zero at Level 2 [20]. This decreases data size, clears space in storage, and improves aggregation. Randhawa et al. in [21] used clustering for aggregation by using fuzzy logic and K-means. Fuzzy inputs includes aggregation rate, utilization of energy, and data persistence, with network lifespan as the consequence. The scheme reduced the duplication ratio. Mohseni et al. in [22] proposed a cluster-based technique with two key steps to enhance energy efficiency and reduce congestion. The initial stage includes clustering sensors. A Capuchin Searching algorithm selects the shortest path between sensors, CH, and BS to reduce energy consumption. The MATLAB simulation shows that the approach improves network longevity, reduces delays, and increases packet delivery rates. The scheme’s Capuchin search algorithm is inefficient for large networks. This study in [23] presents a Qos based multipath routing method designed for IoT applications to overcome single-path routing restrictions such as delays and congestion. The innovative QSBOF improves packet delivery by using metrics such as ETX and workload. Simulation results show a 20% decrease in delay and improvement of 21% in the packet delivery ratio. The system supports a variety of IoT app classes by dynamically assigning pathways based on delay sensitivity. The research emphasizes energy-efficient, low-latency routing methods to increase network stability and performance, while still acknowledging challenges like resource restrictions and scalability.

In [24], A TCP Congestion Control Algorithm (CCA) is provided for usage with IoT devices. When determining new congestion intervals, the algorithm evaluates the network condition and takes into account crucial characteristics. It sets the congestion window using two variables: TCP Bottleneck Bandwidth and Round-trip propagation time (BBR). In cases of high RTT, the proposed algorithm’s throughput may suffer, and this algorithm monitors the rise in end-to-end delay to ensure that the congestion window (CWND) is not altered too quickly, negatively affecting other intra-protocols. The Archimedes method selects active sensors for safe healthcare data transmission, while an attribute-centered binary system chooses the shortest way [25]. Underwater WSNs also undergo clustering and data aggregation [26, 27]. The first layer employs medical devices for tracking the patient’s condition and sends information to the fog server. The fog server prioritizes data based on the health state and severity of the illnesses. This task is performed by fog clusters. If computing expenses surpass the available resources, data is offloaded to the cloud. Finally, reports are generated to help medical staff improve treatment protocols [28]. IoHT combines IoT gadgets, machine learning, cryptography systems and big data, to address issues including cybersecurity, latency, and energy usage. Emerging trends such as monitoring patients remotely and analytics for prediction are critical for improving patient outcomes. Robust encryption techniques, devices authorization, and proactively threat detection are key areas of study. The invention of tiny sensors enables continual tracking of vital signs, hence simplifying early diagnosis. However, problems such as high expenses, handling of data complexities, and privacy concerns persist [29]. The article presents a QoS-aware, multilayered service architecture that uses fog computing to manage IoT data flow efficiently. It solves delay and resource allocation problems by combining multipriority queuing models with a complicated event processing-based scheduling algorithm. The framework greatly improves wait time, utilization of resources, and scalability, demonstrating its capabilities to optimize different IoT services while maintaining QoS under varying traffic situations [30]. This [31] research looks into the use of IoT technology in healthcare, focusing on improvements such as remote patient monitoring, personalized therapies, and effective resource management. It covers issues including privacy of data, interoperability, and standardization. Wearable sensors, telemedicine, and artificial intelligence-powered diagnostics are key applications that, when combined, improve patient involvement, expedite healthcare operations, and reduce expenses through real-time data processing and individualized care methods.

In this category [32], Child node sensor transmit data to their parent nodes, which aggregate the data and transfer it to the central station on the next level, thus building a hierarchy. To properly gather information, the tree-based architecture is employed. Fuzziness applies min-max normalization. A node with a lower weight and direct connection is chosen as a parent. The approach takes into account the complexity of the heterogeneous environment, but there is no mechanism for controlling energy use as attributes rise. A PQTBA is suggested in [33] to reduce network congestion in Internet of Things networks. The PQTBA prioritize network traffic based on real-time requests using a preemptive/non-preemptive approach and a discretionary rule. In terms of data throughput, packet loss ratio, and energy consumption, the proposed approach performs better than current methods. IoT networks experience congestion as a result of growing traffic rates, which raises packet drop due to buffer occupancy.

The resource limits of wearable devices for health monitoring suffer from limited computation power and energy. The mobile edge computing (MEC) support the extensive processing resources to process the data closer to the source. It increases response time and alleviates network congestion. Priority-based systems which assign work based on emergency levels, have received attention in research. These strategies ensure that high-priority jobs, especially in latency-sensitive healthcare applications, are handled rapidly while balancing system resource utilization and decreasing total processing time and bandwidth costs presented in [34]. IoT systems use smart sensors and wearable devices to collect real-time data on parameters such as heart rate, body temperature, and physical activity. Machine learning techniques, such Decision Trees and Naïve Bayes, are used to evaluate data and identify health hazards. This strategy enables proactive care, particularly for older adults living alone, which improves both health outcomes and quality of life [35].

The main goal of the study in [36] is to address the issue of buffer overflow, which causes packet loss in environments with limited resources, in 6LoWPAN-based IoT healthcare networks. An analytical model, which calculates the likelihood of packet loss because of buffer overflow, is proposed. The study illustrates the impact of congestion on IoT-healthcare network performance, highlighting the unsuitability of conventional congestion control techniques for the unique requirements of healthcare applications. The results illustrate packet drop rates and buffer loss probability, offering guidance on how to efficiently handle congestion in these kinds of networks. In [37] main emphasis is on the critical need for appropriate scheduling in IoT-based applications for healthcare, where delays can have significant consequences, including death of patients. The study proposes a superior scheduling system, termed to be the Prioritized Scheduling (PS) scheme, which is a variation on the Earliest Deadline First (EDF) scheduling method. It is intended to directly address the weaknesses of EDF in cases where it is overwhelmed. The scheme’s limitations and drawbacks include the complexity of implementation, the overhead of classification, and scheduling. A method for congestion-aware data transfer on mobile and limited IoT networks is proposed. To address the congestion problem, the study in [38] proposes a technique for congestion-aware data transfer which uses Hop-to-hop communication to maintain congestion awareness and efficiency, and several parameters are also considered before selecting the next hop. The proposed method uses the Analytic Hierarchy Process (AHP) to make multi-parameter-based decisions in order to determine the optimal next hop. For IoT-enabled WSNs, in healthcare environments, where sensor nodes gather patient physiological data. The study in [39] suggests a distributed congestion control algorithm (DCCA). The study emphasizes how network performance can be negatively impacted by congestion, resulting in packet loss and decreased reliability. Priority-based routing and congestion control are combined in the suggested method to guarantee the timely transmission of vital healthcare data. The model’s efficiency in avoiding congestion and enhancing the network’s reliability for applications related to healthcare is validated by simulations.

**R{3**,**1} The author in** [40] **discussed the queue aware congestion avoidance (QACA) scheme**,** which uses controlled acknowledgement for the congestion avoidance. The suggested framework shares the queue status with the sender nodes via a controlled acknowledging-based approach in order to reducing the congestion. Until a specific percentage of the queue is not filled**,** such as more than 50%**,** the intermediate node initially does not report the queue status. When the queue reaches 50**,** 70**,** or 90%**,** the technique takes into account the three probable rates for transmitting the acknowledgment packet every 10**,** 5**,** or 1 packet. By adjusting the data rate**,** congestion can be promptly identified and potential congestion can be avoided but the scheme did not discussed the data duplication issues. In** [41] **the author discussed the priority-based congestion avoidance scheme (PBCAS)**,** where incoming data is classified into critical and non-critical categories. Packets in the critical group are routed through the shortest path using SVM classification**,** while TOPSIS is used for routing non-critical data. The primary focus of the study was on prioritizing packets into high- and low-priority queues; however**,** the proposed scheme did not address data de-duplication**,** which may result in increased delay and network congestion.**

According to the literature assessment, majority queue-based congestion avoidance approaches continuously send redundant data to intermediate nodes. This technique places additional processing load on these devices and contribute considerably to network congestion. By delivering repeated data, the schemes in [33, 36, 37, 39] fail to optimize resource consumption, resulting in inefficiencies in data transmission and lower overall network performance. In D-QACA, the congestion avoidance is better as compared to other schemes but this scheme also not focusing on the de-duplication of the redundant data. It is also mentioned that the delay in processing of emergency packets in IoHT can have serious repercussions. In the literature, the base schemes are categorizing the packets into high and low priority but not processing as per the emergency level of the packets.

## System model and problem statement

This section discusses the system model, which includes a smart health-monitoring system for early congestion detection in network queues and packet-loss prevention for emergency messages. The study’s purpose is to develop a priority-based queueing technique for IoT-based health monitoring systems that can handle packets containing emergency data. Congestion arises in emergencies when a large volume of messages is sent between nodes and the gateway. Even when congestion increases, the model ensures that emergency packets are delivered on time.

This system model is made up of four sorts of nodes: (i) leaf nodes, which are sensor devices that include both wearable and non-wearable sensors. The mentioned sensing devices are used to obtain data from the patient’s body. **R{1**,**6}**,** R{1**,**5} (ii) The collector node**,** whether mobile or dedicated computational equipment**,** receives packets from the network**,** classifies them as high or low priority**,** checks for the duplication of the data**,** and Boolean substitution. (iii) Intermediate nodes are medical objects with significant computing and network resources; the intermediate nodes process data collected from sensing devices based on the packet’s priority and transmit it to the local sink node for further processing. (iv) Local sink nodes are fog servers with enormous computational power; the sink node’s primary function is to preprocess packets received from intermediate nodes**, i.e.,** to figure out the type of healthcare services needed by a patient by examining packet data; and iv) the root node**,** which is the cloud.** The cloud is linked to the hospital’s administrative system, which medical professionals use.

**Fig. 1:**
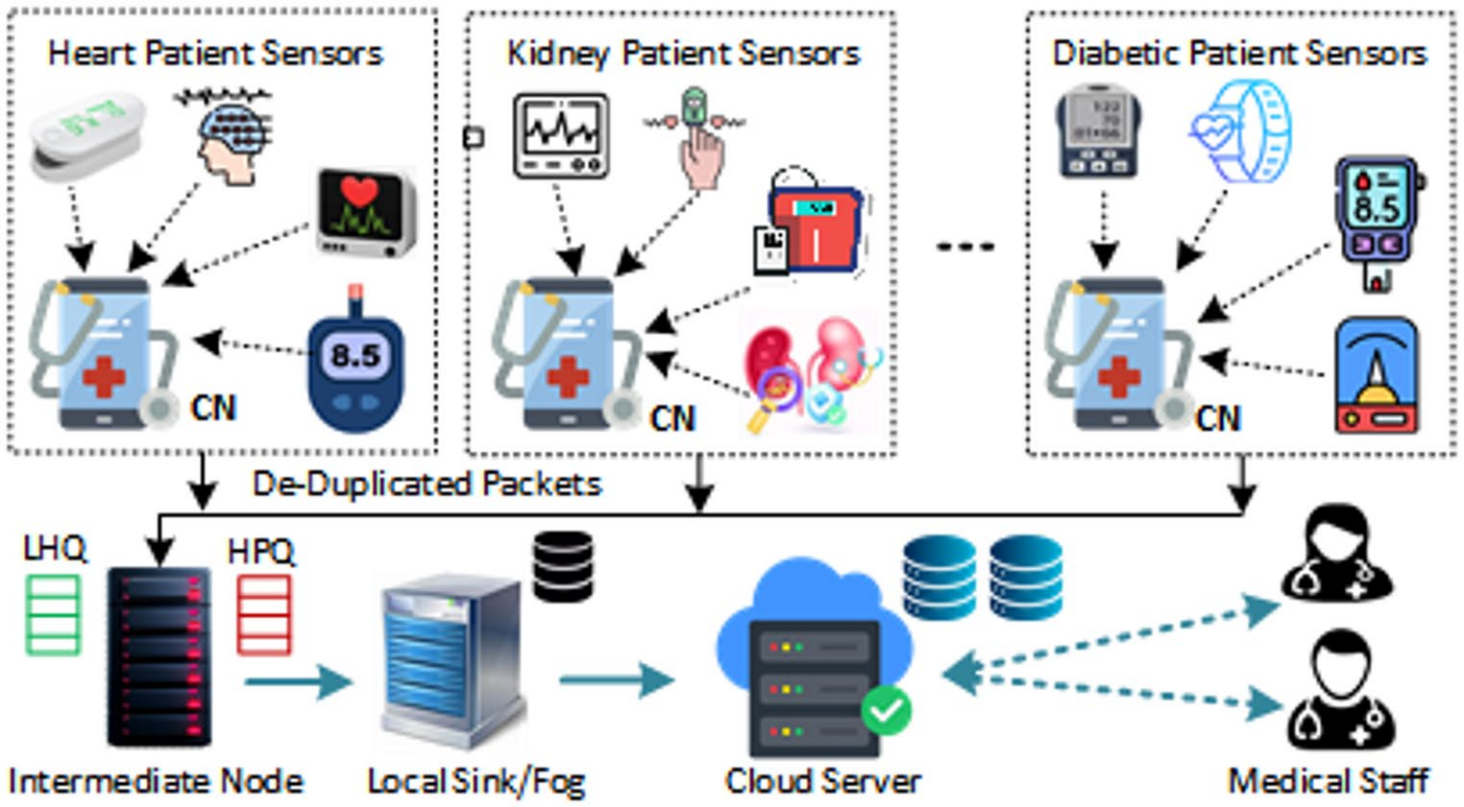
Proposed system model for healthcare data sharing

IoHT sensing devices, including smartwatches, smartphones, and other gadgets, serve as leaf nodes. Nowadays, electronic watches and smartphones are frequently used as leaf nodes to route sensing data to local sink nodes via collectors and intermediary nodes. This processed information is transferred to the cloud servers (root nodes) via the network, as presented in Fig. 1.

The main problem is that the sensors that are deployed at Level 1 in healthcare monitoring systems receive and send physiological values from the patients to intermediate nodes without filtering out duplicate or redundant information. At the intermediate node, incoming data can be handled utilizing high- and low-priority queues to distinguish between critical and non-critical data. A large percentage of these measurements are within the normal range, indicating stable, non-threatening conditions. Sending such redundant information in its complete 16- or 32-bit form consumes more energy from the sensors and increases communication costs throughout the network. This inefficiency not only shortens the operating lifespan of the sensors, but it also causes network congestion due to filling of high and low priority queues, which can delay the transfer of crucial data.

### **R{3**,**2} Challenges and practical considerations**

**The various practical challenges faced by the proposed system warrant consideration during real-life implementation. One of the major issues is the selection and setting of clinically appropriate threshold values (THL and THU)**,** which require close coordination with established medical standards to reduce the risk of misclassifying abnormal values. The other challenge is heterogeneous sensor data: different physiological sensors generate data at different rates and with varying payload sizes**,** which may disrupt queue dynamics when traffic is high. Scalability is also an issue**,** as the development of monitored patients and devices is bound to put heavy strain on both collector and intermediate nodes. In addition**,** the system should be resilient to changing network characteristics**,** such as varying traffic demands and network disconnections**,** and ensure that emergency packets are sent with minimal delay. Lastly**,** the collector node faces configuration and administration overhead**,** as it must re-examine incoming data and maintain correct priority and emergency tagging without adding excessive processing delay.**

## De-duplication and queue-usage aware congestion avoidance scheme

This section describes the proposed “De-duplication and Queue-usage Aware Congestion Avoidance (DD-QACA) scheme to solve the identified problem.” The suggested system employs a priority-based queue, with each node having two buffer queues. Each queue has a different priority level: low and high. When the node gets an input packet, it categorizes it and delivers it to its appropriate queues. The packets in the high- and low-priority queues are not at the same level and have different levels of emergency. In the healthcare sector, a number of sensing devices have been embedded in patients’ bodies to collect various types of data. These measurements vary because sensors that collect single-part numbers, such as BPM, return 16-bit data. Similarly, some sensors turn readings into two values. For instance, sensor blood pressure tracking generates data in two formats: systolic and diastolic values. Both produce 16-bit data, totaling 32 bits. It creates a highly diverse environment, which boosts energy use.

**Fig. 2:**
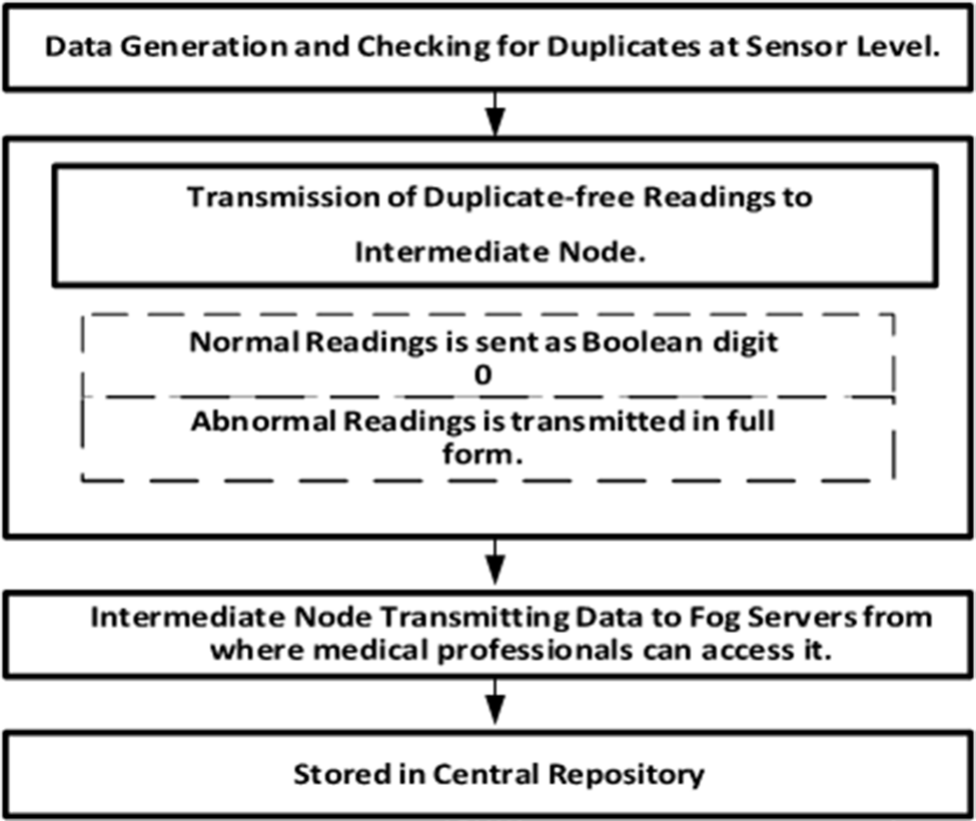
Steps of de-duplication

**R{1**,**4} At level 1**,** the suggested approach checks whether the sensor data falls within the typical range. In the suggested DD-QACA model**,** THL and THU denote the lower and upper limits of the clinical threshold used to define the normative operating range for each physiological parameter. Measurements in the closed interval [THL**,** THU] are considered stable and can be prepared by Boolean substitution; measurements that are not in this interval are considered abnormal**,** and they are sent in full fidelity such as body temperature 36–38 °C**,** heart rate 60–100 bpm**,** SpO2 no less than 92**,** and blood pressure within the accepted limits of adult human ranges. Such thresholds can be configured and adjusted in accordance with existing clinical guidelines or deployment instructions.** Suppose it falls within the usual range, rather than delivering the full 16 or 32 bits. Only the Boolean digit 0 is sent, requiring only one bit, as shown in Fig. 2.

Furthermore, our proposed method is adaptable to threshold changes because the intermediary node combines and processes data within the specified threshold ranges. The specified ranges can be easily adjusted at any time by the intermediary nodes. Sensors record patient body readings as concatenated strings. For example, patient A reported the following data for the body’s temperature, pulse, oxygen level, cholesterol levels, and sugar level: 98.9:75:98:130:180. Data obtained from the body is graded as normal or crucial. In an average person, such parameter readings are consistent and within normal limits. In these circumstances, the senior nodes just convey a single Boolean digit 0 to the intermediate node. On the other hand, if an individual is ill, irregularities may be visible in one or two sensor readings, whereas the rest of the values often remain unchanged. In such cases, the crucial data is sent in its original form, while the remaining sensors transmit only a Boolean digit. In extremely rare instances, all installed sensors generate unexpected data. In such instances, all values are transmitted in their original format. The list of notations used in this work is shown in Table 1.

**Table 1:** List of notations

Sr.	Notation	Description
1.	$r-node$	Root Node
2.	$i-node$	Intermediate Node
3.	*LPQueue*	Low Priority Queue
4.	*HPQueue*	High Priority Queue
5.	*lc_node*	Lowest cost node
6.	*Packet*	Packet Received from the root node
7.	*QSBOF*	Quality-of-Service Balanced Objective Function

### Queuing at intermediate node

The queues are maintained at the intermediate node, where packets are categorized as high or low priority depending on the type of the sensor. These packets are processed based on the critical level and priority assigned to them. Packets containing sensitive information, such as heartbeat or brain signals, are prioritized and processed based on the severity of emergency. Packets with less sensitive data are placed in a low priority queue.

The system indicates that sensors produce patient data packets, which are then sequentially passed to a collector node, with each packet assigned a priority and emergency level. The system features two queues, one for high-priority packets and one for low-priority packets, such that packets of greater urgency are handled first. If the two packets have the same emergency level, the first to arrive is given priority. The system assumes that processing is non-preemptive, which implies that existing packets are processed before a new packet is treated. The suggested queue management strategy will dynamically change the high-priority packets by placing them at an index adjacent to the packet currently in process.

The model runs with a finite buffer capacity, highlighting efficient management to avoid overflow. These assumptions provide a realistic and solid foundation to assure system reliability in the context of healthcare.

### Duplicate reading removal algorithm

The DD -QACA functionality is presented in Algorithm 1, entitled the Duplicate Reading Removal Algorithm (DRRA). It involves handling emergency packets, placing them in the queue first to ensure they are delivered on time with minimal delay. }**R{2**,**1} To formally describe the decision logic of Algorithm** 1:

$$xi\left( t \right) = \left\{ {\begin{array}{*{20}{c}} {0} \\ {ri\left( t \right)} \end{array}} \right.\begin{array}{*{20}{c}} \begin{gathered} if\,THLi \leq ri\left( t \right) \leq THUi\, \hfill \\ and\,emergency = 0 \hfill \\ \end{gathered} \\ {otherwise} \end{array}$$

The algorithm takes the form of steps 1–14, in which the input is a set of healthcare sensor nodes, which are represented by: S =(1, n2, n3,..., n14). Readings are collected by each sensor node, processed, and stored. The result will be two data strings: the processed data from high-priority and low-priority sensors are stored in dataStringHPQ and dataStringLPQ, respectively. The three string variables aggrString, dataStringHPQ, and dataStringLPQ are initially initialized to the empty string. The counter variable count is set at 0 to count the received readings or iterations. The measurements taken by each sensor node are produced within a set time frame of time period t, and each sensor node provides the measurements in each sensor node as a set of readings in n[i].readings[]. The algorithm checks the emergency flag (n[i].emergency_flag) and the full data flag (FData Flag); both are flagged clear. When these conditions are satisfied, the system will repeat the process of processing all the sensors’ readings, i.e., The sensor nodes, denoted by a sensor number n[i], verify their priority flag; when the sensor node is a High Priority one (n[i].flag = High Priority), the readings that it has processed, namely, through the AggregateMessage function, are concatenated into the dataStringHPQ with a delimiter DL2 between values of different sensors. If the sensor is marked as Low Priority, its readings are processed and concatenated into dataStringLPQ using the same delimiter.

In steps 15 to 27, the algorithm increments the count variable by 1 to monitor the number of sensor readings aimed at. At a specified count limit, the count is reset to zero, and the FData Flag is set to true, allowing full sensor values to be delivered rather than truncated values. When this requirement is met, one repeats the process for all sensors (k) and appends the entire readings to aggrString. The concatenation of the values is done with the delimiter, \(DL1:(:)<|human|>. The values are concatenated to show the sensor readings for each node. After reviewing all the readings, the algorithm assigns a priority level to each sensor. In order to keep data of high-priority sensors in the high-priority queue, the delimiter of the sensor or the delimiter of the sensor readings, which is a dash, such as the delimiterDel2dep608DL2(“_”), is appended to the sensor readings as the concatenated length of the sensor readings to form the data string, which is high priority, i.e. the dataStringHPQ. On the other hand, when the sensor is of Low Priority, its aggrString is concatenated into dataStringLPQ using the same delimiter, preserving the data’s proper classification and structure. The sensor readings are then processed using the AggregateMessage function between steps 28 and 35 before they are stored. The function takes IN_ARGS as input, where IN is a sensor node reading. It initially compares each reading value with the lower threshold THL and the upper reference threshold THU to determine whether it falls outside the predefined interval. The delimiter (DL1:) of readings into the delimiter (:) is used to concatenate the actual into aggrString so that the values of the reading would be separated correctly in case the reading is considered abnormal (less than THL or more than THU). To minimize redundancy and improve data transmission speed, the function uses a Boolean value of 0 to extend the end of aggrString rather than storing the actual value when the reading falls within the normal range.

**Figure:**
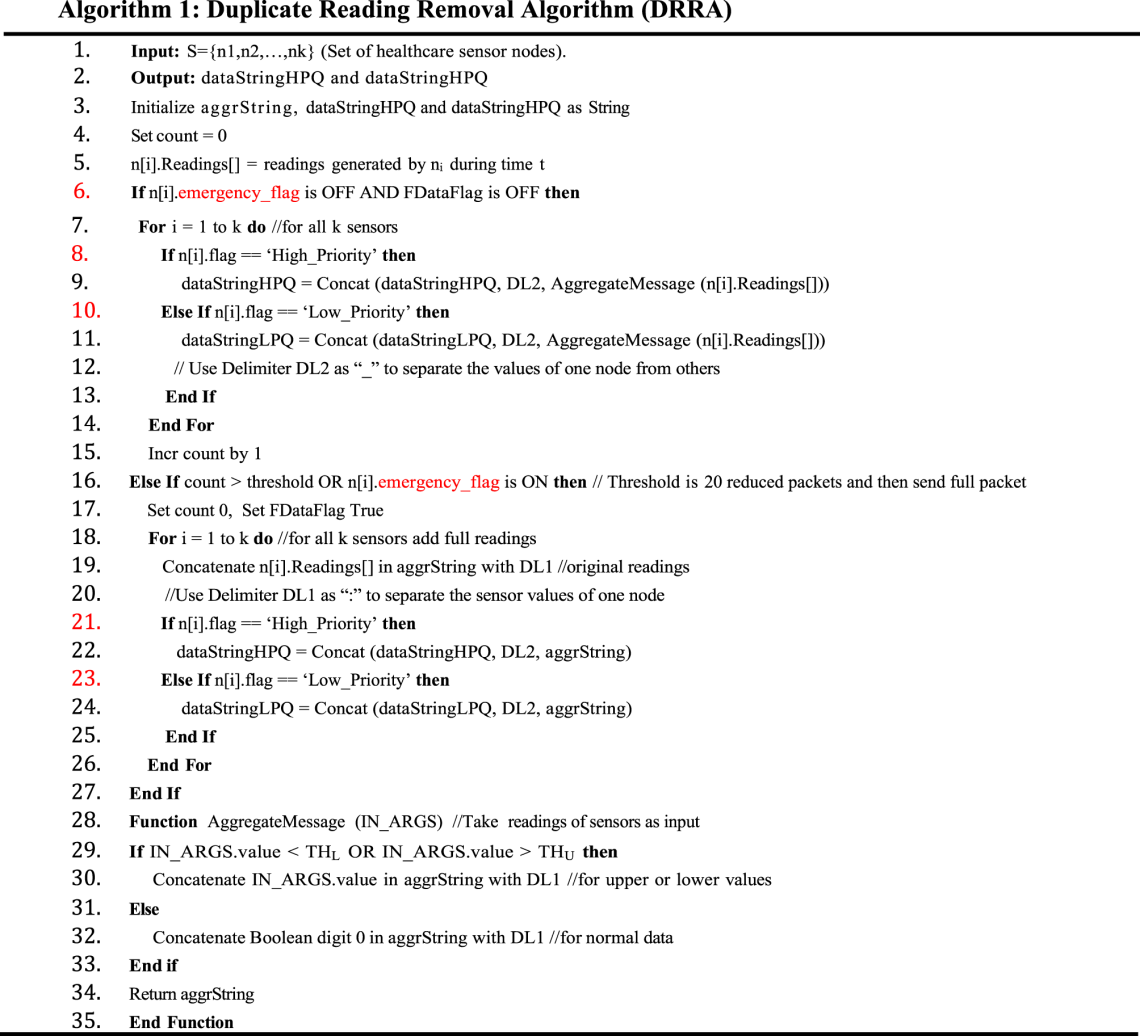

The intermediate node receives collected information from collector nodes and also verifies the status of the emergency flag, which, if true, indicates that the person being monitored is in critical condition and the data is forwarded to high or low priority queue as per the nature of the sensors. In the priority queue, each packet is prioritized based on the severity of emergency. If no other packets are being processed, the packet containing the emergency data is given the greatest priority and is processed first. If a packet is currently being processed, it is stored in the second place.

## Result and analysis

In NS2.35, extensive simulations were conducted to evaluate the effectiveness of the proposed DD-QACA method. The sensor nodes were used in a 500 m x 500 m mesh, with a nominal transmission range of 30 m that was kept the same regardless of the type of feature. Data packets were transmitted every 30 s. A TCL script was used to fully specify the network parameters, making it easier to configure and deploy network nodes. Packet transmission and reception functionality were implemented using special C + + classes. Lastly, the AWK scripts were used to process and extract the obtained data. In a traditional IoT-based hospital monitoring system, sensors (including electrocardiographs (ECGs), blood-pressure devices, pulse oximeters, thermometers, accelerometers, and electroencephalographs (EEGs)) continuously collect patient data and send it to a collector node (CN). The CN then collects this information and sends it to an intermediary node (IN), which, in turn, forwards it to a centralized server, where it is further processed and analyzed. In an emergency, all sensor data streams are relayed; each patient produces large amounts of data, which are sent to the intermediate node every 15 s. An example is an ECG sensor with a 250 Hz sampling rate and 16-bit resolution, which translates to 500 bytes per second, about four kbps. A blood-pressure meter with 16-bit resolution and a 1-second sample rate generates 2 bytes/s (an average of 16 bps). An oxygen saturation pulse oximeter (SpO2) at 1 Hz with 16-bit resolution produces 4 bytes per second (32bps). The rate of a temperature sensor with a 12-bit sampling rate at a 1-second sampling frequency is 2 bytes/second (= 16 bps). A tri-axial accelerometer/gyroscope that measures motion at 50 Hz with 16-bit resolution of three axes yields 300 Bytes per second (2.4 kbps). The EEG sensor is the most data-intensive, with 500 samples/s at 24 bits of resolution across eight channels, yielding 12,000 bytes/s (about 96 kbps). In aggregate, all vital signs send about 106.4 kbps of information to the CN. The effectiveness of the proposed model was compared with that of other schemes. The parameters used in the evaluation are listed in Table 2.

**Table 2:** Simulation parameters with respective values

Parameters	Values
Simulation Duration Deployment Area Number of Nodes Data Packet Size No. of Simulations Time Consumed Number of Packets Channel Capacity	1500 s 500 × 500 60 500 bits 15 0–30 s 0–50 150 kbps & 250 kbps
Maximum Packets in Queue	50
Organization of nodes	Random
Sensing Radius	30 m

### Buffer loss probability

**Fig. 3:**
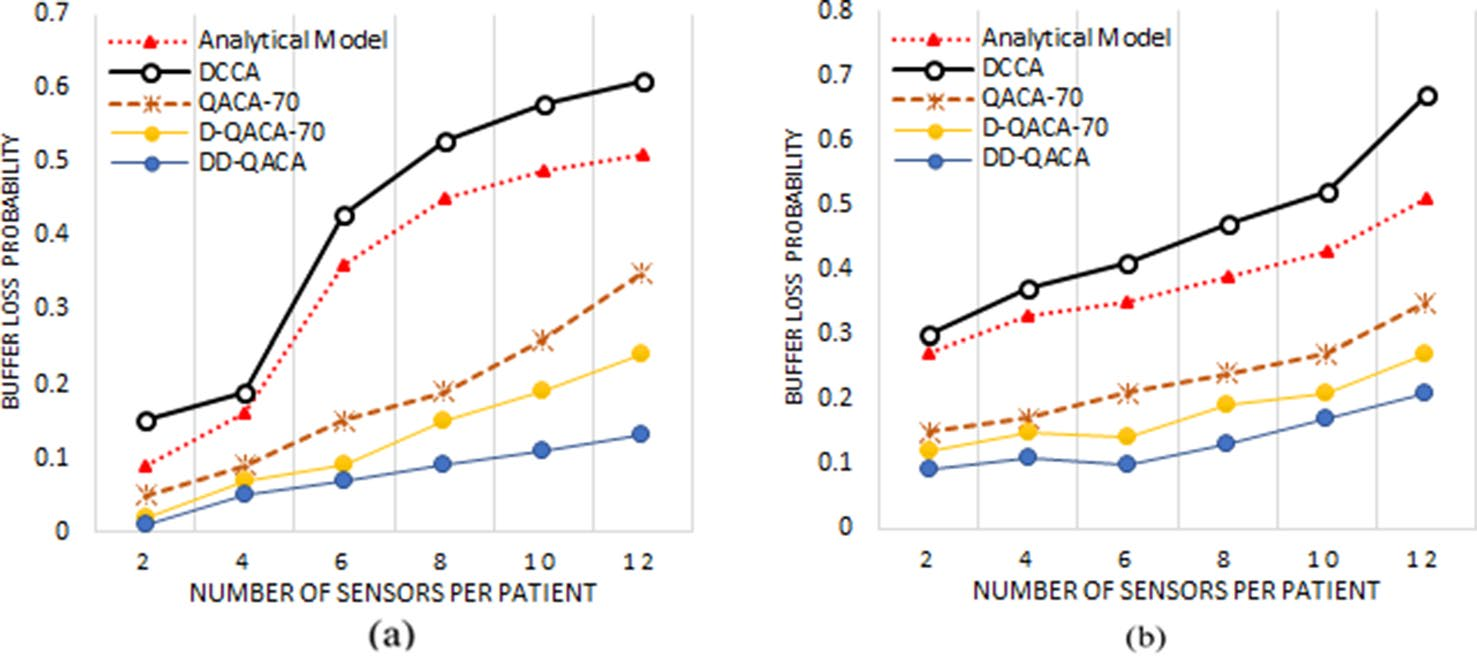
Buffer loss probability for (**a**) Intermediate nodes having a channel Capacity of 250 kbps whereas (**b**) represent the same for 120kbps

Figure 3 illustrates the performance by considering channel capacity of 250 kbps and 120 kbps. It reduces buffer loss probability while optimizing the processing of emergency packets. Figure 3(a) illustrates 36%, 43%, 15%, 9% and 7% for Analytical Model, DCCA, QACA-70, D-QACA-70, and proposed DD-QACA, respectively when 6 sensors are attached per patient. In this scenario, there were 10 CNs and each patient with 06 sensors results into data from 60 sensors, which results in increasing packet size and buffer loss ratio. Similarly, 4(b) present the buffer loss probability for intermediate nodes for a channel capacity of 120 kbps.

Overall, it has been observed that the buffer loss probability in the Analytical Model, DCCA, QACA-70 and D-QACA-70 remains high due to the frequent transmission of duplicated sensor information to intermediate nodes. Such limitations cause a lot of data gathering in network buffers, enhancing the probability of packet loss. The proposed DD-QACA achieves better buffer utilization by managing packet size and delivering full values only in emergencies and Boolean flag values for unaltered data. At intermediate nodes, whenever a queue is idle, emergency messages are sent to higher indices or handled directly which improving responsiveness. proposed system for emergency packets is nearly nil, even when a queue is 90% full at intermediate and leaf nodes. The packets are only eliminated from the queue tail, whereas emergency packets are immediately placed at the first index, next to the current packet in process.

### High priority packet lost per second

**Fig. 4:**
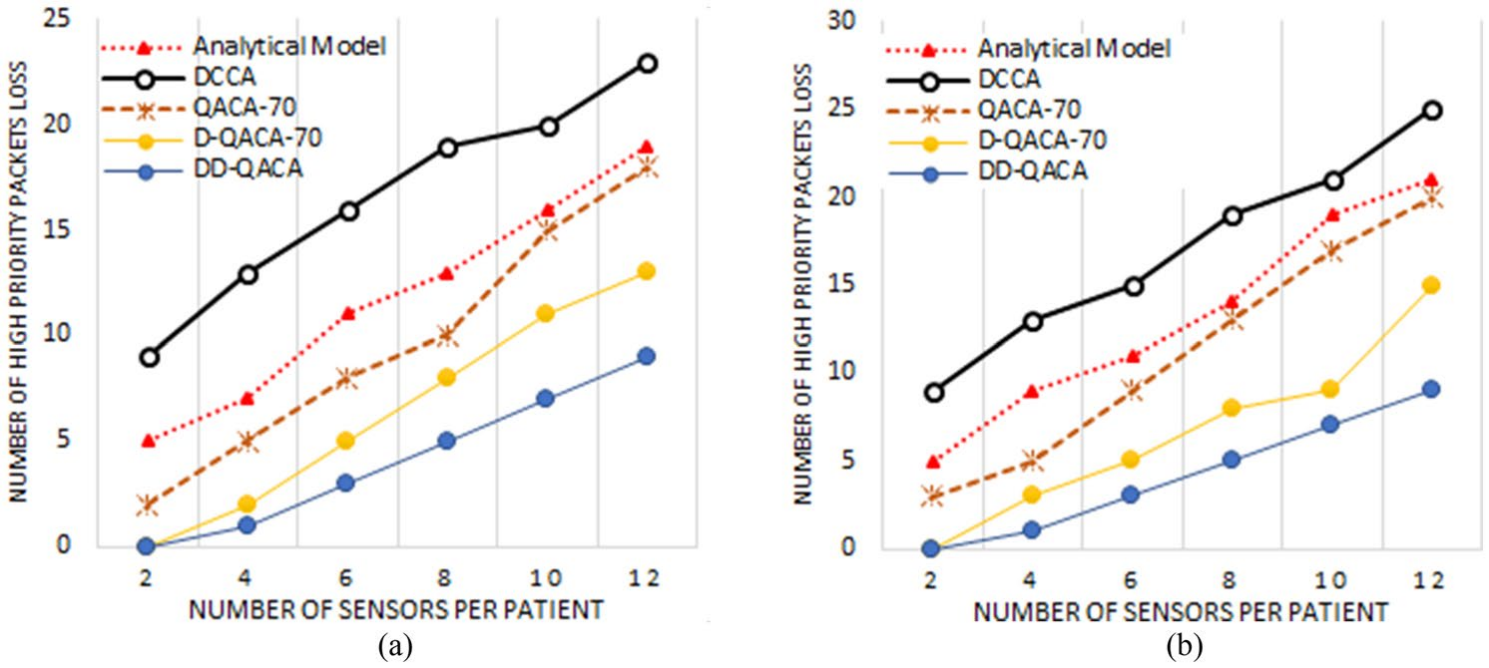
High priority packet lost for (**a**) Intermediate node having a channel Capacity of 250 kbps and (**b**) represent the same for 120kbp

Figure 4 illustrate the packet loss probability for different models at intermediate nodes for 250 kbps and 120 kbps channel capacities with changing numbers of nodes. Figure 4(a) shows that the Analytical Model has the largest packet loss, increasing from 5 packets at 2 nodes to 19 packets at 8 nodes and up to 23 packets at 12 nodes due to continual full-data transmission and acknowledgment overhead. DCCA also suffers high packet loss, beginning at 9 packets at 2 nodes, increasing to 19 packets at 8 nodes, and peaking at 23 packets at 12 nodes, because it broadcasts full readings and only send acknowledgment when congestion occurs. QACA-70, which optimizes acknowledgments, has lower packet loss than these models, but it still loses two packets at two nodes, ten packets at eight nodes, and 18 packets at twelve nodes. D-QACA-70 decreases packet loss by prioritizing vital data, resulting in losses of 0 packets at 2 nodes, 8 packets at 8 nodes, and 13 packets at 12 nodes. DD-QACA has the lowest packet loss, keeping 0 packets at 2 nodes, 5 packets at 8 nodes, and 9 packets at 12 nodes, because of to its flag-based transmission technique that reduces congestion. Similarly, Fig. 4(b) shows the high priority packet loss of intermediate nodes with a channel capacity of 120 kbps.

### Packets delay

**Fig. 5:**
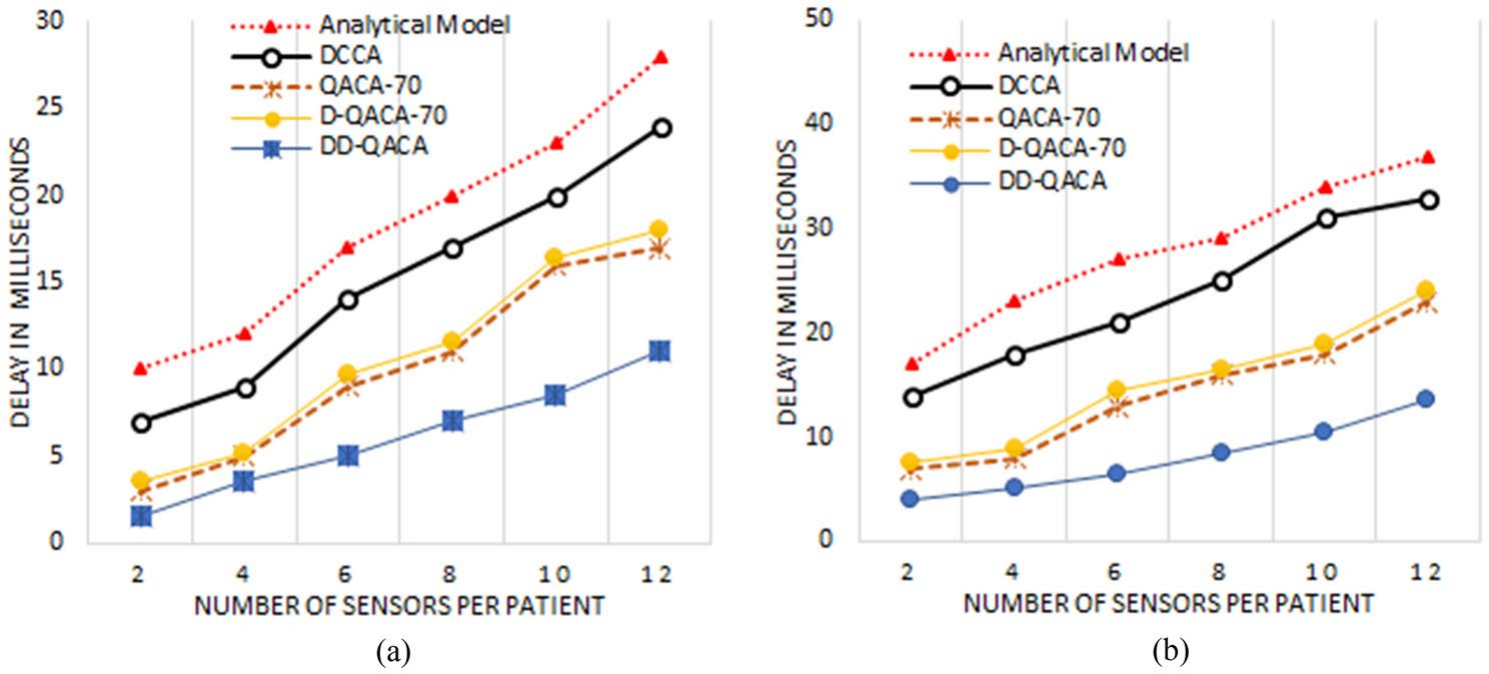
Delay of Packets for the channel (**a**) Capacity 120 kbps and (**b**) 250 kbps

Figure 5(a), illustrate the packet delay across the different models with the channel capacity of 250 kbps and 120 kbps at the intermediate node. Figure 5(a) illustrate that 17, 14, 9, 9.7 and 5 for Analytical Model, DCCA, QACA-70, D-QACA-70 and DD-QACA respectively when the sensors attached to the patient body are six. DD-QACA consistently displays the lowest delay, demonstrating its efficiency. Figure 5(b) show the same with the channel capacity of 120 kbps at the intermediate node. The proposed DD-QACA scheme’s reduction in packet size is critical for reducing the transmissions delays. DD-QACA differs from Analytical, DCCA, QACA-70, and D-QACA-70 in that it dynamically changes packet length by substituting regular data with Boolean digits (0) and only transmits full values in emergencies. This significantly reduces packet size, allowing for quicker data transmission and fewer network queuing delay. The rate of packet reduction in size is determined by the number of readings replace with Boolean values. If 50% of sensor readings are changed, the overall packet size is decreased by roughly 48%; replacing 70% of readings results in a 67% reduction. In an ideal scenario where all normal readings are replaced by Boolean values, the packet size can be reduced by up to 95%, significantly cutting communication overhead and significantly reduce packets delays. For example, in a standard approach where each full data reading costs 32 bits (4 bytes), transferring 10 sensor readings would ordinarily take 320 bits (40 bytes). In DD-QACA, replacing 50% of the readings decreases the packet size to 165 bits (~ 21 bytes) and 70% to 103 bits (~ 13 bytes). By sending solely Boolean values, the packet size can be as tiny as 10 bits (~ 2 bytes), significantly reducing transmission overhead.

### Communication cost

**Fig. 6:**
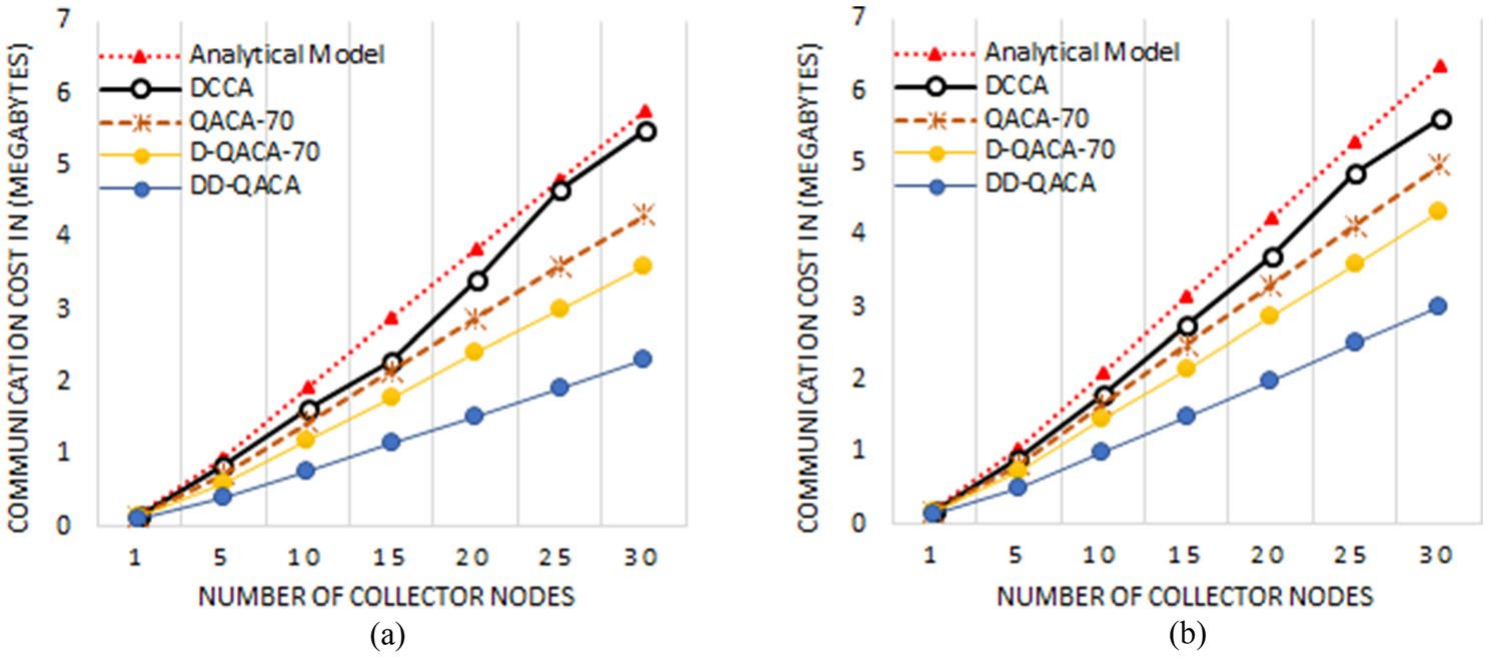
Communication cost for the channel (**a**) Capacity 120 kbps and (**b**) 250 kbps

Figure 6 illustrate the communication cost in bytes for several schemes using 250 kbps and 120 kbps channel capacity and varying the number of collector nodes. Figure 6(a) shows the 250 kbps scenario for the intermediate node, higher bandwidth enables smoother data delivery, resulting in cheaper communication costs. The analytical model faces the highest communication cost because it transmits entire data as well as acknowledgments including queue status. For instance, at 15 collector nodes, the analytical model costs 2.8 MB. DCCA also sends complete readings but reduced cost of 2.3 MB. QACA-70 reduces the number of acknowledgements while still delivering full readings, lowering costs to 2.16 MB. D-QACA-70 significantly prioritizes high-priority packets, reducing the cost to 1.8 MB. DD-QACA has the lowest cost by simply transmitting flag bits during normal circumstances and sending complete data in emergencies or after a predetermined time, resulting in a cost of 1.15 MB. Figure 6(b) illustres the 120 kbps case, the lesser bandwidth causes congestion, resulting in increased communication costs for all models. The analytical model costs 3.16 MB, whereas DCCA is close behind at 2.78 MB. QACA-70 reduces the cost to 2.48 MB, whereas D-QACA-70 further improves data transfer, yielding 2.16 MB. DD-QACA maintains the most efficient, with a transmission cost of just 1.49 MB. The suggested DD-QACA technique reduces communication costs by transmitting simply flag bits in normal conditions while sending all data only during emergencies or at predetermined intervals, resulting in fewer needless transfers. In contrast, some methods, such as the Analytical Model and DCCA, communicate entire data every time, resulting in higher communication cost because of frequent transmissions and acknowledgements. Furthermore, QACA-70 and D-QACA-70 limit acknowledgments and prioritize vital data while still transmitting complete readings, resulting in greater communication costs than DD-QACA.

## Conclusions and future work

This study presents the DD-QACA scheme, a novel way of improving the efficiency of high-priority data transmission in networks. The technique reduces buffer loss by using flag values for sensor readings that are within normal range. It also ensures that emergency packets are processed on time by placing them at higher indices or immediately if no other packets are currently being handled. By eliminating redundant data transfer, the technique optimizes the bandwidth and storage in the collector node. Whenever sensor readings remain constant, simply a flag is delivered rather than the entire data value, which saves network resources. This optimization becomes increasingly visible as the number of sensors increases. Conventional models have more packet loss owing to congestion. NS 2.35 is used for simulation and TCL file contains the node configuration, deployment of the nodes, message initiation, and trace annotation, and the results show that buffers loss probability for the proposed scheme in case of emergency packets has been reduced by 90% when compared to base schemes. This ensures that essential data is less likely to be lost caused by buffer overflow, enabling the system more trustworthy in addressing emergency situations. Furthermore, the probability of emergency packet loss has been reduced by 95%, indicating that critical data packets are more likely to reach the collector node successfully, improving the system’s overall reliability. The limitations of this work include the energy consumption for sending all readings of sensors, although replacing duplicate values with Boolean flags massively reduce communication cost and energy utilization. Secondly, a little more energy is consumed to perform computations for replacing Boolean values but this cost is negligible as compared to saving in communication cost. Future work will consider the fuzzy logic for reducing the duplication in case of large data at higher devices to further reduce the storage cost at central repositories as well.

## Data Availability

No new data were generated or analyzed in this study.
